# Supplementary material for: Integrative proteomics reveals principles of dynamic phosphosignaling networks in human erythropoiesis
Source: Mol Syst Biol. 2020 Dec 1;16(12):e9813. doi: 10.15252/msb.20209813 (PMC7706838; doi:10.15252/msb.20209813)
Supplement: Supplementary file 1 — Expanded View Figures PDF [file MSB-16-e9813-s001.pdf]

Expanded View Figures

Figure EV1. Establishing stage-specific proteomes of erythropoiesis.

A FACS gating/sorting regime to enrich for CD235a⁻ progenitor population.
B Characterization of the differentiation stages in culture. May–Grünwald–Giemsa staining of erythroid cells is shown. Scale bar, 20 μm.
C Coefficient variations (CVs) of four biological replicates for each protein were calculated in all stages to show the reproducibility of our system. Dashed line shows the cutoff line of 20% CV.
D Cumulative protein abundance and dynamic range in five differentiation stages. Hemoglobin subunits (HBB, HBA1, HBE1 and, HBG1) are labeled as progenitor (yellow) and Ortho (orange) stages.
E Estimated median copy numbers of histones per cell across all measured stages.

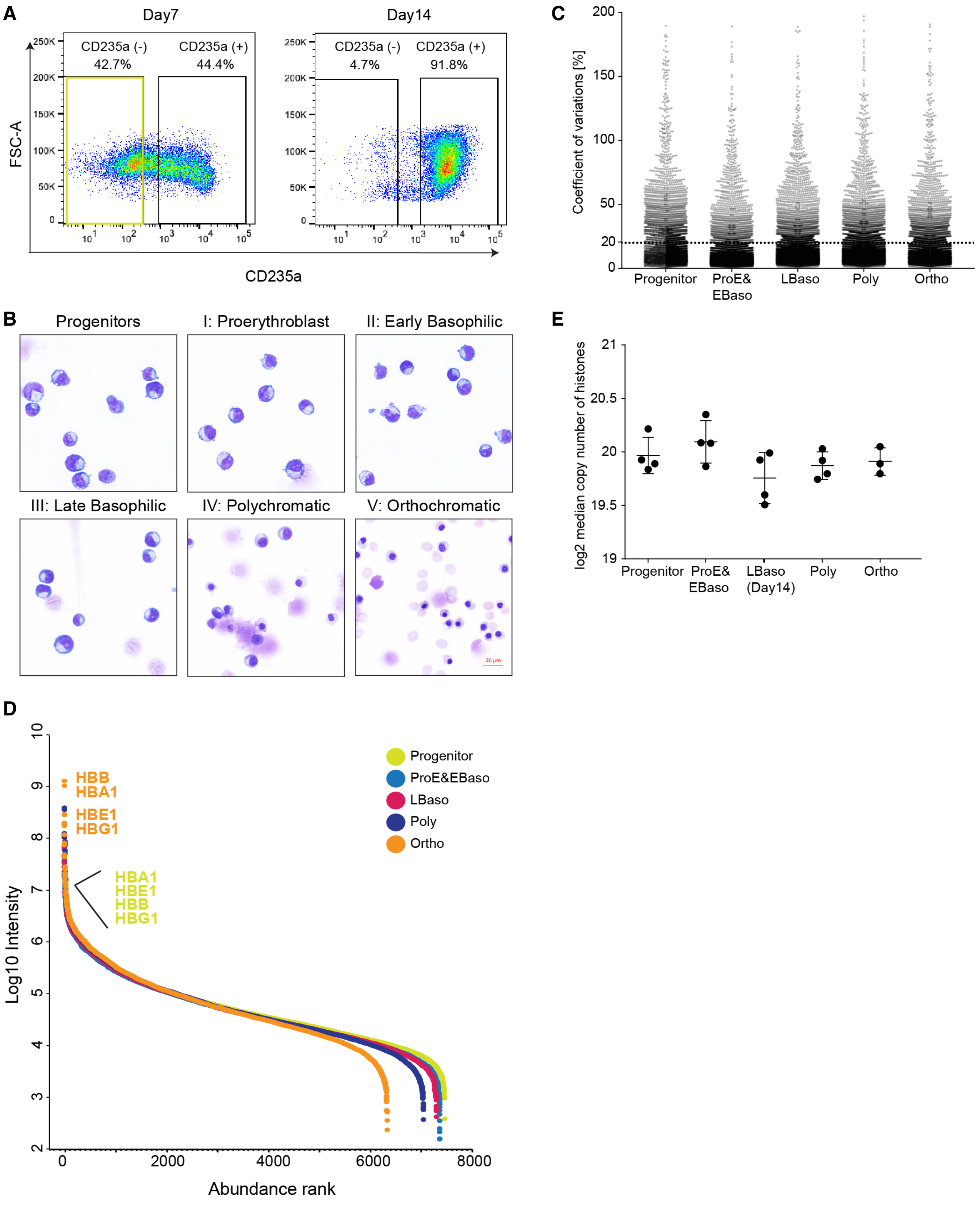

Figure EV1.

 © 2020 The Authors

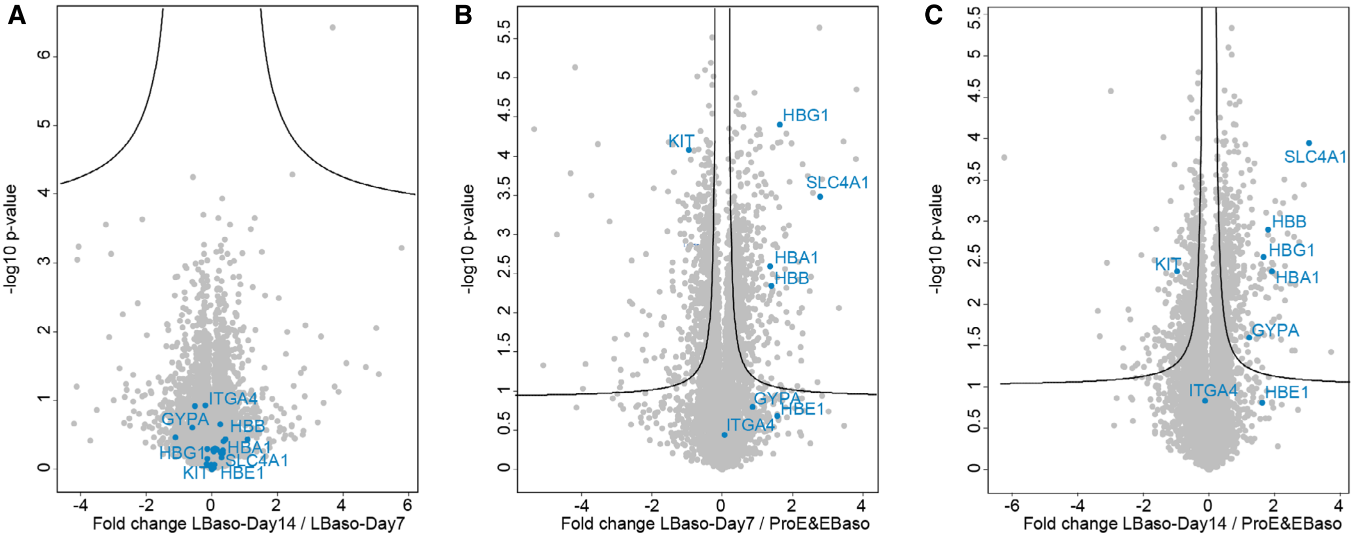

Figure EV2. Comparison of differentiation stage-specific proteomes of human erythropoiesis.

A–C Volcano plots of the (-log10) *P*-values versus the log2 protein abundance differences between LBaso day 7 and LBaso day 14 (left), LBaso day 7 and ProE-EBaso (middle), and LBaso day 14 and ProE-EBaso (right) with the significance lines (FDR < 0.05 and S0 = 0.1). Selected marker proteins are labeled in blue.

Figure EV3. Gene Ontology (GO) enrichment analysis of significant proteome clusters.

Gene Ontology (GO) enrichment analysis of six clusters of significant proteome shown in Figure 2A was performed using Fisher's exact test. 2% threshold was applied to Benjamini–Hochberg FDR to determine the significance.

▶

© 2020 The Authors

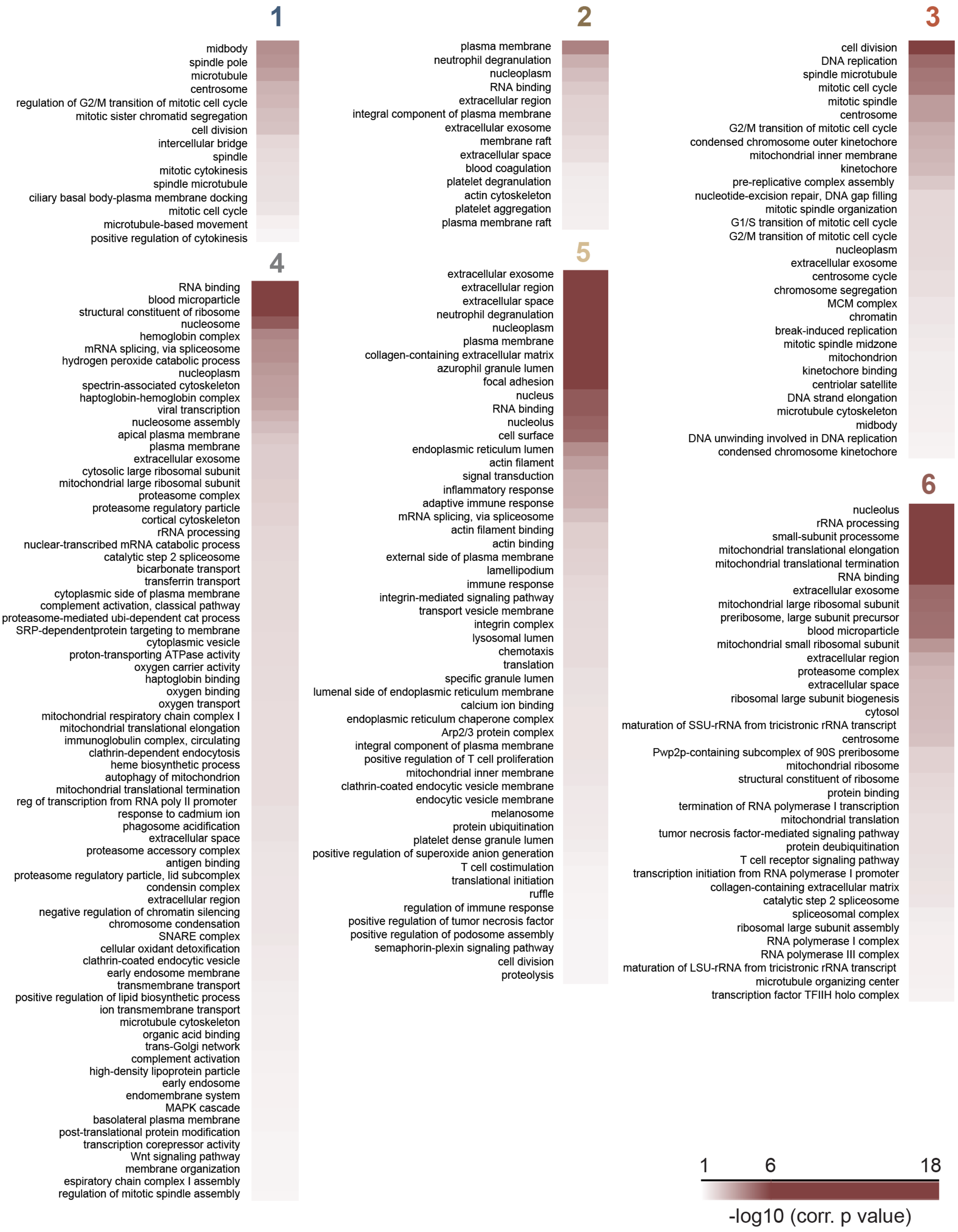

Figure EV3.

© 2020 The Authors

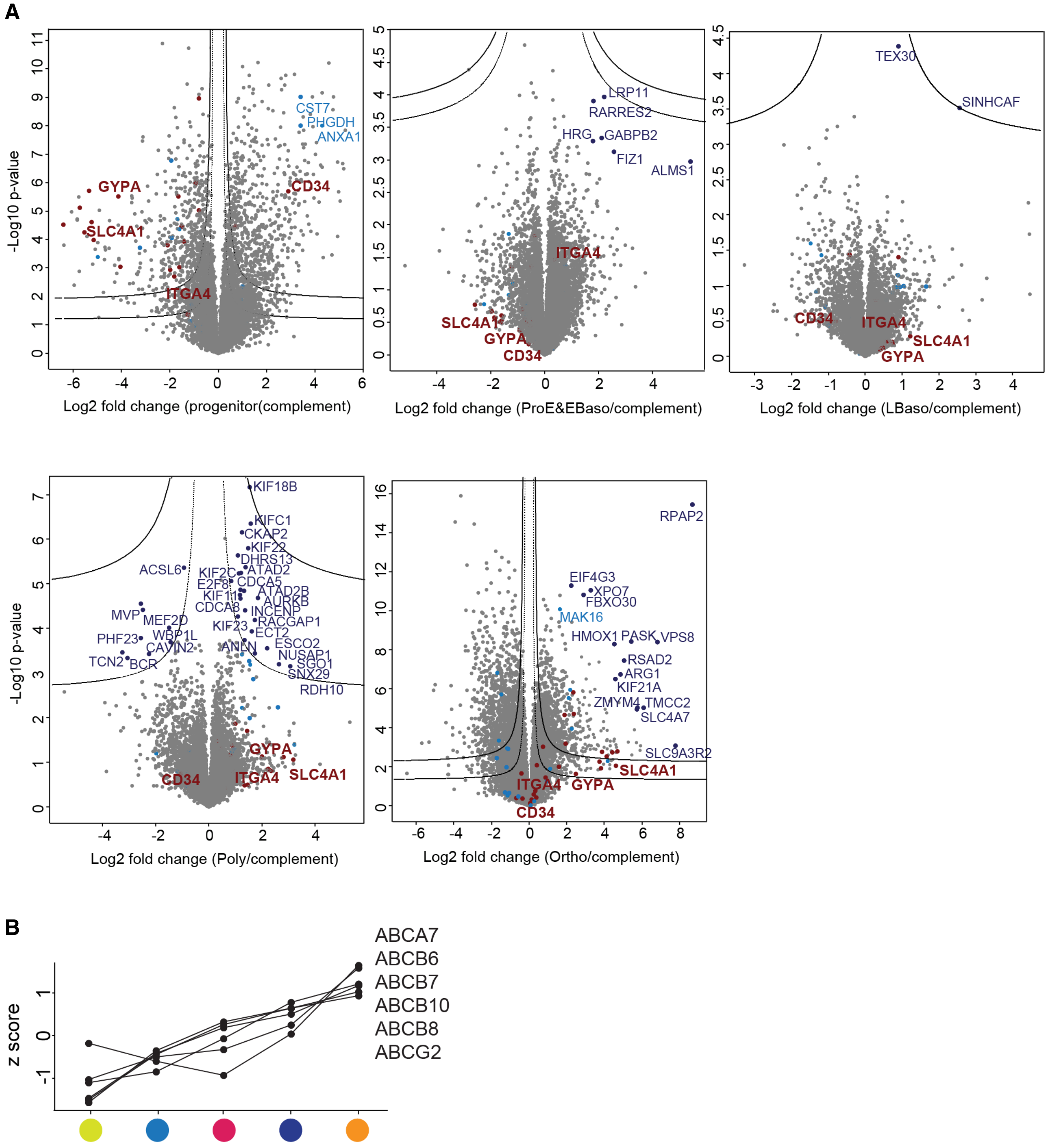

Figure EV4. Stage-specific significantly regulated proteins.

A, B Hawaii plots that overlay all volcano plots of protein enrichments in a specific stage over all other stages plotted against corresponding *P*-values. Two cutoff lines were placed graphically, defining two confidence classes with FDRs of 0.01 and 0.05 (S0 = 0.1). Sorting and cluster markers, and selected outliers are labeled in dark red, light blue, and dark blue, respectively.

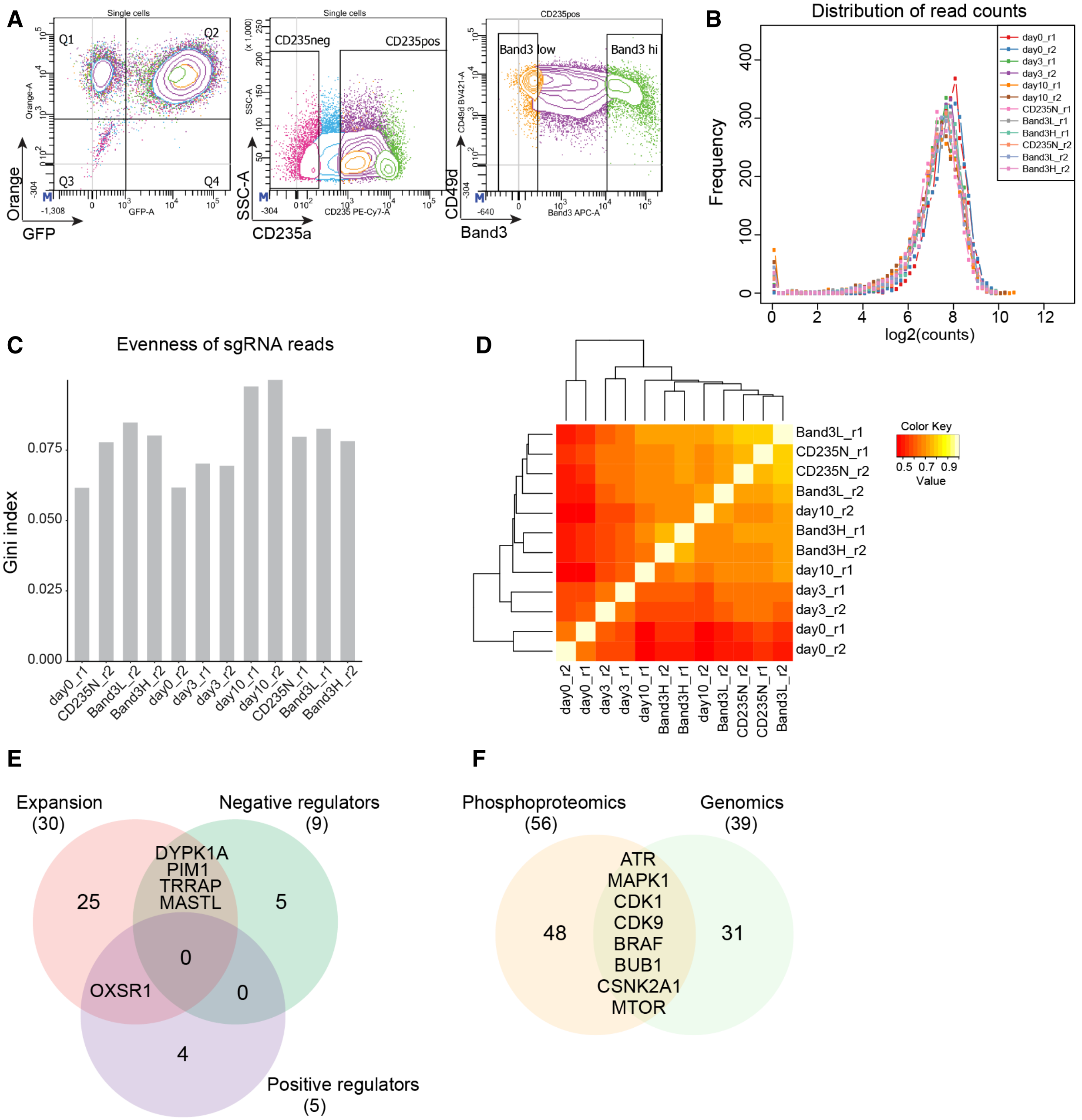

Figure EV5. Establishing kinome-targeting CRISPR/Cas9 screen in HUDEP-2 cells.

A Flow cytometry strategy based on GFP, CD235a, CD49d, and Band3 to determine the significant hits from the genome-scale CRISPR-Cas9 screen.
B Histogram of the sgRNA distribution in each sample in the CRISPR-Cas9 kinase screen.
C Evenness of the sgRNA reads in each sample in the CRISPR-Cas9 kinase screen.
D Correlation based reproducibility analysis between replicates in the CRISPR-Cas9 kinase screen. High and low correlation values are denoted in yellow and orange, respectively.
E Overlap between expansion hits and positive or negative maturation regulators.
F Overlap of kinases whose activities were inferred by stage-specific substrate profiling from phosphoproteomics in Figure 4F and the genomic CRISPR-Cas9 kinase screen.

 © 2020 The Authors

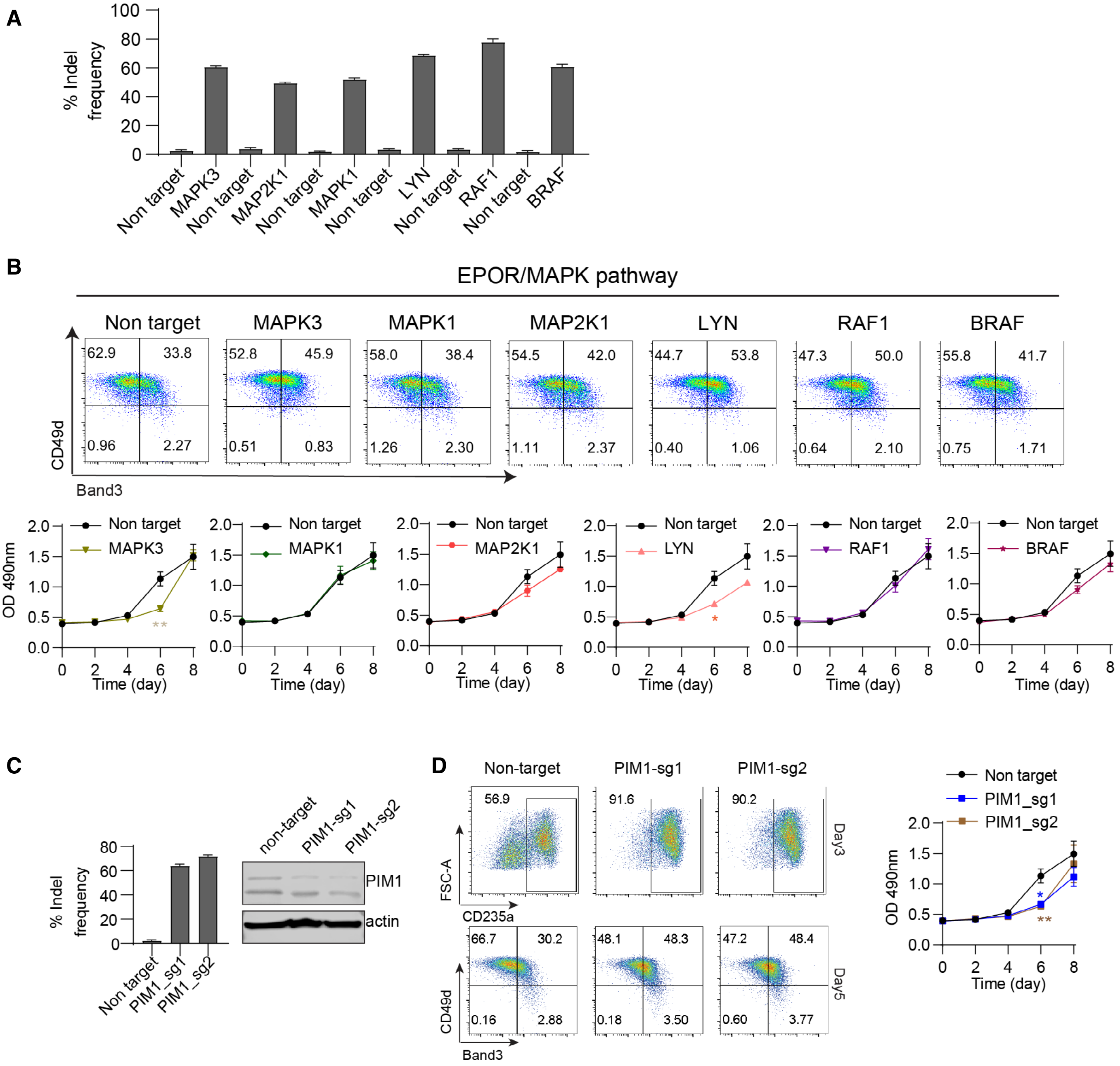

Figure EV6. Validation of candidate kinases.

A Indel frequencies at 3 days after transduction of Cas9-expressing HUDEP2 cells with lentiviral vector encoding individual sgRNAs targeting the indicated genes.

B Top panels show FACS analysis of Band3 and CD49d expression in HUDEP2 cells after Cas9 + sgRNA disruption of the indicated genes followed by culture for 3 days in differentiation medium to induce terminal erythroid maturation. The bottom panels show cell numbers measured by a colorimetric CellTiter Glo assay of gene-targeted cells grown in expansion medium, with the light absorbance (OD 490nm) readout shown on the y-axis. Data show the results as the mean ± SEM for three biological replicate experiments. *$P < 0.05$; unpaired *t*-test.

C Cas9-expressing HUDEP-2 cells were transduced with lentiviral vector expressing PIM1-targeting or control non-targeting sgRNAs and cultured for 3 days. Left panel shows indel frequencies determined by PCR followed by next-generation sequencing. Right panel shows Western blot analysis of whole cell lysates using β-actin antibody as a loading control.

D Left panels show FACS analysis of Band3 and CD49d expression in HUDEP2 cells after Cas9 + sgRNA disruption of *PIM1* followed by culture for 3 days in differentiation medium to induce terminal erythroid maturation. The right panels show viable cell numbers measured by a colorimetric CellTiter Glo assay of gene-targeted cells grown in expansion medium, with the light absorbance (OD 490nm) readout being shown on the y-axis. Data show the results as the mean ± SEM for three biological replicate experiments. *$P < 0.05$, *$P < 0.01$; unpaired *t*-test.

© 2020 The Authors
